# Soy Glycinin Contains a Functional Inhibitory Sequence against Muscle-Atrophy-Associated Ubiquitin Ligase Cbl-b

**DOI:** 10.1155/2013/907565

**Published:** 2013-05-25

**Authors:** Tomoki Abe, Shohei Kohno, Tomonari Yama, Arisa Ochi, Takuro Suto, Katsuya Hirasaka, Ayako Ohno, Shigetada Teshima-Kondo, Yuushi Okumura, Motoko Oarada, Inho Choi, Rie Mukai, Junji Terao, Takeshi Nikawa

**Affiliations:** ^1^Department of Nutritional Physiology, Institute of Health Biosciences, The University of Tokushima Graduate School, 3-18-15 Kuramoto-cho, Tokushima 770-8503, Japan; ^2^Medical Mycology Research Center, The University of Chiba, Chiba 260-8673, Japan; ^3^Division of Biological Science and Technology, College of Science and Technology, Institute of Biomaterials, The University of Yonsei, Wonju 220-710, Republic of Korea; ^4^Department of Food Science, Institute of Health Biosciences, The University of Tokushima, Tokushima 770-8503, Japan

## Abstract

*Background*. Unloading stress induces skeletal muscle atrophy. We have reported that Cbl-b ubiquitin ligase is a master regulator of unloading-associated muscle atrophy. The present study was designed to elucidate whether dietary soy glycinin protein prevents denervation-mediated muscle atrophy, based on the presence of inhibitory peptides against Cbl-b ubiquitin ligase in soy glycinin protein. *Methods*. Mice were fed either 20% casein diet, 20% soy protein isolate diet, 10% glycinin diet containing 10% casein, or 20% glycinin diet. One week later, the right sciatic nerve was cut. The wet weight, cross sectional area (CSA), IGF-1 signaling, and atrogene expression in hindlimb muscles were examined at 1, 3, 3.5, or 4 days after denervation. *Results*. 20% soy glycinin diet significantly prevented denervation-induced decreases in muscle wet weight and myofiber CSA. Furthermore, dietary soy protein inhibited denervation-induced ubiquitination and degradation of IRS-1 in tibialis anterior muscle. Dietary soy glycinin partially suppressed the denervation-mediated expression of atrogenes, such as MAFbx/atrogin-1 and MuRF-1, through the protection of IGF-1 signaling estimated by phosphorylation of Akt-1. *Conclusions*. Soy glycinin contains a functional inhibitory sequence against muscle-atrophy-associated ubiquitin ligase Cbl-b. Dietary soy glycinin protein significantly prevented muscle atrophy after denervation in mice.

## 1. Introduction

Skeletal muscle atrophy caused by unloading is characterized by both decreased responsiveness to myogenic growth factors, such as insulin-like growth factor (IGF-1) and insulin, and increased proteolysis [1–3]. We reported previously that unloading stress resulted in skeletal muscle atrophy through the induction and activation of a ubiquitin ligase Casitas B-cell lymphoma-b (Cbl-b) [4]. Cbl-b induced ubiquitination and degradation of insulin receptor substrate-1 (IRS-1), the IGF-1 signaling intermediate. In turn, the loss of IRS-1 caused the forkhead transcription factor-3- (FOXO3-) dependent induction of muscle atrophy F-box protein (MAFbx)/atrogin-1, a dominant mediator of proteolysis in atrophic muscle. We also demonstrated that Cbl-b-deficient mice were resistant to unloading-induced atrophy and loss of muscle function [4]. These results indicate that the Cbl-b-dependent degradation of IRS-1 is a critical mediator of increased protein degradation and reduced protein synthesis in unloading-induced muscle atrophy.

We have also reported that a pentapeptide mimetic of tyrosine^608^-phosphorylated IRS-1, DGpYMP, which we named Cblin (Cbl-b inhibitor), prevented denervation-induced muscle atrophy in mice [4]. We also found that Cblin inhibited Cbl-b-mediated IRS-1 ubiquitination and expression of MAFbx/atrogin-1 [4]. These results indicated that the inhibition of Cbl-b-mediated IRS-1 ubiquitination could be therapeutically beneficial in unloading-induced muscle atrophy.

Soy protein and derived peptides are effective for mitigation of muscle damage [5], promotion of protein synthesis, and translation initiation after exercise [6]. In addition, we demonstrated previously that diet containing soy protein prevents exercise-induced protein degradation in skeletal muscle, through the suppression of calpain-mediated proteolysis [7]. Thus, dietary soy protein affects protein turnover in skeletal muscle. However, to date, there is no information on the effects of soy protein on unloading-induced muscle atrophy.

In a preliminary study, we found that the sequence of soy glycinin, a major component of soy protein, is similar to that of Cblin. Based on this finding, we explored whether soy glycinin and its derived peptides prevent denervation-associated muscle atrophy in mice. Treatment of HEK293 cells with soy-glycinin-derived peptides suppressed Cbl-b-mediated IRS-1 ubiquitination. Furthermore, mice fed with soy glycinin showed inhibition of denervation-associated muscle atrophy, and this effect was mediated through the conservation of IGF-1 signaling. These findings indicated that soy glycinin is a useful dietary protein for muscle atrophy associated with muscle unloading.

## 2. Materials and Methods

### 2.1. Multiple Alignment of Amino Acid Sequence

We analyzed the primary structure of glycinin precursor with ClustalX software (http://www-igbmc.u-strasbg.fr/BioInfo/ClustalX/Top.html). 

### 2.2. Cell Culture

HEK293 cells were maintained in Dulbecco's modified Eagle medium (DMEM) containing 10% fetal bovine serum, 100 U/mL penicillin, and 100 *μ*g/mL streptomycin at 37°C under 5% CO_2_-195% air gas mixture.

### 2.3. Transfection

HEK293 cells (40–60% of confluence) were transfected with plasmid vectors, using Hily Max lipofection reagent (DOJINDO, Kumamoto, Japan), as described previously [8]. The expression plasmids used in this study were pCEFL-human Cbl-b-HA, pcDNA3.1-rat IRS-1-V5, and pcDNA3-FLAG-Ubiquitin.

### 2.4. Immunoblotting and Immunoprecipitation

Immunoblot and immunoprecipitation analyses were performed as described previously [9]. The following antibodies were used: anti-actin (Calbiochem, La Jolla, CA, USA), anti-HA.11 (BabCo, Richmond, CA, USA), anti-V5 (Invitrogen, Carlsbad, CA, USA), anti-FLAG M2 (Sigma, St. Louis, MO, USA), anti-IRS-1 (Upstate Biotechnology, Lake Placid, NY, USA), anti-Akt-1 (PharMingen International, Tokyo), and anti-phospho-S^473^-Akt-1 (Cell Signaling Technology, Beverly, MA, USA).

### 2.5. Cell-Free Ubiquitination Assay

The cell-free ubiquitination assay was performed as described previously [4]. We prepared immunoprecipitated Cbl-b (IP-Cbl-b) and IRS-1 (IP-IRS-1), respectively, bound to anti-HA- and anti-IRS-1-IgG-linked protein A beads from extracts (1 mg protein) of HEK293 cells transfected with pCEFL-Cbl-b-HA and pcDNA3.1-IRS-1-V5 (5 *μ*g). Slurries of IP-Cbl-b and IP-IRS-1 were incubated at 37°C for 4 hours in reaction buffer (50 *μ*L) containing ATP-regenerating system, recombinant mouse E1 (500 ng), UbcH7 (5 *μ*g; E2), GST-tagged ubiquitin (10 *μ*g), and/or ubiquitin-aldehyde (Ubc-CHO) (1 *μ*g; a deubiquitinase inhibitor), which were purchased from Boston Biochem Inc. (Cambridge, MA, USA). Then, 20 *μ*L of boiled supernatant was separated by SDS-PAGE and visualized by immunoblotting with an anti-IRS-1 antibody.

### 2.6. Preparation of Soy-Derived Peptides and Synthetic Peptides

We used the following pentapeptides or hexapeptides derived from soy: soy-glycinin-derived peptide, soy-*β*-conglycinin-derived peptide, lipoprotein- (LP-) derived peptide, and soy protein isolate (SPI). These peptides were kindly provided by Fuji Oil Co. (Osaka, Japan). In addition, we used the following synthetic peptides: Cblin, DGpYMP, which exhibited inhibitory activity on Cbl-b-mediated IRS-1 ubiquitination in our previous study [4]. A patent application was lodged for the sequence of this peptide (Pat. no. 5113346). Negative control was a peptide with VGpYLR sequence, which had no inhibitory activity against Cbl-b-mediated IRS-1 ubiquitination [4]. These peptides were kindly provided by Otsuka Pharmaceutical Co. (Tokushima, Japan). We also synthesized a pentapeptide with Cblin-like amino acid sequence in soy glycinin, termed it Cblin-like peptide or DIpYNP, and checked its inhibitory activity using cell-free ubiquitination assay, as described above. Since soy-glycinin-derived peptides showed inhibitory activity on IRS-1 ubiquitination in the cell-free system (pat. no. 4963044), it was subjected to further analysis in cell cultures.

### 2.7. Animal Models

Experiments were conducted in C57BL/6J mice (Japan SLC, Shizuoka, Japan). In anesthetized 6-week-old mice, the sciatic nerve of the right leg was cut and a 10 mm piece was excised. The left leg remained innervated and was used as a control. Mice were sacrificed at 1, 3, 3.5, or 4 days after operation. Mice were fed one of the following four diets from 1 week before denervation till sacrifice: a mixed diet containing 20% casein protein (Control diet); a mixed diet containing 20% SPI (SPI diet); a mixed diet containing 20% soy glycinin protein (20% glycinin diet); and a mixed diet containing 10% casein protein plus 10% soy glycinin protein (10% glycinin diet). These proteins were kindly provided by Fuji Oil Co (Osaka, Japan). The macronutrient compositions of the different diets are listed in Table 1. The Committee for the Care and Use of Laboratory Animals of The University of Tokushima approved all protocols of this study, which were conducted according to the Guide for the Care and Use of Laboratory Animals at The University of Tokushima (http://cms.db.tokushima-u.ac.jp/DAV/organization/10998/anex-HP/anex/aigo-t/tokusima-u_doubutukannrikisoku.pdf).

### 2.8. Histochemical Analysis

The tibialis anterior (TA) muscle was isolated after sacrifice and immediately frozen in chille disopentane and liquid nitrogen and stored at −80° as described previously [10]. Sections were stained with hematoxylin and eosin (H&E). TA muscle sections were digitally captured using bright field with BZ-II Analyzer (KEYENCE, Osaka, Japan). Digital imaging was performed at 10x final magnification. Image processing was performed using Adobe Photoshop CS (Adobe Japan, Tokyo). The circumference of each fiber was outlined using an ImageJ software (National Institute for Health) to generate CSA of myofibers. Criteria used in the selection of muscle fibers to measure for CSA of myofibers included an intact, distinct cell membrane without significant signs of distortion or folding. Elongated fibers indicating an oblique section were excluded. Image analyses were performed by two coauthors (TY and TS).

### 2.9. Real-Time RT-PCR

To measure the amount of mRNA in small samples, real-time reverse transcription-polymerase chain reaction (RT-PCR) was performed with SYBR Green dye using an ABI 7300 real-time PCR system (Applied Biosystems, Foster City, CA, USA) as described previously [11]. The oligonucleotide primers used for amplification are listed in Table 2.

### 2.10. Statistical Analysis

All data were statistically evaluated by multiple comparison using Excel Statistic ver. 6.0 (Esumi Co, Tokyo, Japan) and were expressed as mean ± SD. Differences between two groups were assessed with Duncan's multiple range test or Shirley-Williams' multiple comparison test. Differences were considered significant at *P* < 0.05.

## 3. Results

### 3.1. Identification of Cblin-Like Sequence in Soy Glycinin

**Figure 1:**
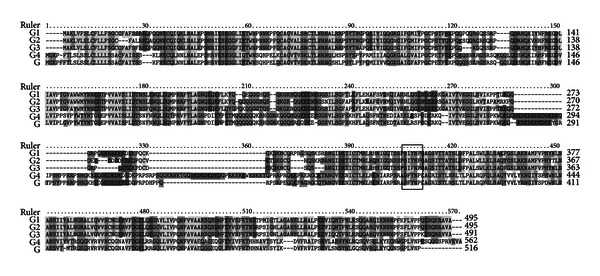
Alignment of glycinin precursor protein sequences. The sequence of soy glycinin was similar to that of Cblin peptide, indicated by the box. The sequences were retrieved from the UniProt database (http://www.uniprot.org/). G1, Glycinin G1; G2, Glycinin G2; G3, Glycinin G3; G, Glycinin G; G4, Glycinin G4.

We reported previously that Cblin, DGpYMP, inhibited Cbl-b-mediated ubiquitination and degradation of IRS-1 both *in vitro* and *in vivo* [4]. We searched proteins containing Cblin-like sequence using the Basic Local Alignment Search Tool (http://blast.ncbi.nlm.nih.gov/Blast.cgi/) and found that soy glycinin in soy protein contains a sequence similar to that of Cblin peptide, DI/FYNP. Multiple sequence alignments of five soy glycinin homologues, G1, G2, G3, G4, and G, indicated that Cblin-like sequence, DIYNP, is conserved among these glycinin homologues (Figure 1).

### 3.2. Cblin-Like Peptide in Soy Glycinin Inhibits Cbl-b-Mediated IRS-1 Ubiquitination in HEK293 Cells

**Figure 2:**
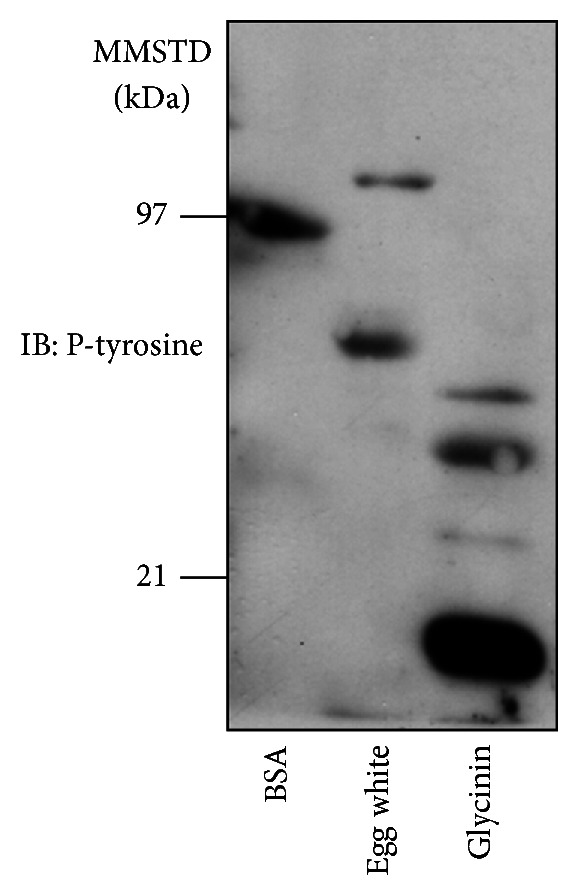
Identification of phosphorylated tyrosine in soy glycinin. Bovine serum albumin, egg white, and soy glycinin were subjected to immunoblot analysis for phosphorylated tyrosine. MMSTD: molecular mass standards. Representative findings of three experiments with matching results.

**Figure 3:**
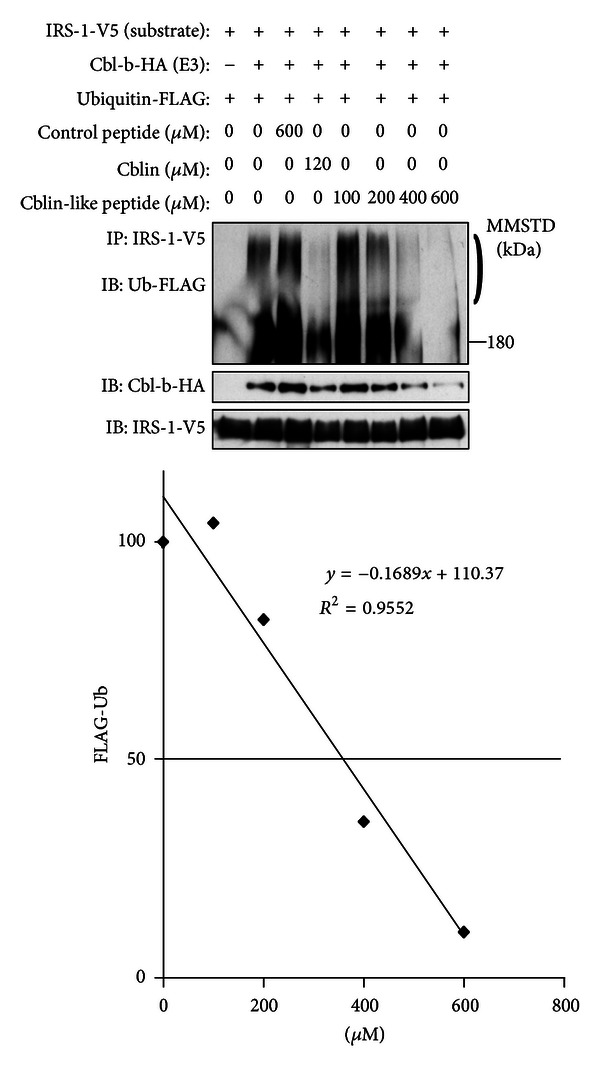
Inhibitory effect of Cblin-like synthetic peptide on Cbl-b-mediated degradation and ubiquitination of IRS-1. HEK293 cells were transfected with mock vector/pCEFL-Cbl-b-HA, pcDNA3.1-rat IRS-1-V5, and pcDNA3-FLAG-Ubiquitin and then treated with 100 nM epoxomicin at 2 hours before treatment with each peptide at the indicated concentrations. One hour later, these cells were treated with 10 ng/mL IGF-1 for 1 hour. Immunoprecipitates (IP) from whole cell lysates incubated with an anti-IRS-1 antibody were subjected to immunoblot (IB) analysis for the indicated proteins. The densitometric measurement of FLAG-Ub (the area indicated by right parenthesis) was performed with an ImageJ software (National Institute for Health). MMSTD: molecular mass standards. Representative findings of three experiments with matching results.

First, we synthesized pentapeptides of the above sequence, including Cblin-like synthetic peptide, DIpYNP, and DIYNP. West and Towers [12] reported that food-derived phosphopeptides were absorbed, at least in part, without dephosphorylation. Immunoblot analysis showed that soy glycinin contained more phosphorylated tyrosine than egg white (Figure 2). Therefore, we used the phosphorylated peptide, DIpYNP, in the following experiments. Treatment with IGF-1 induced ubiquitination of IRS-1 in HEK293 cells transfected with Cbl-b, IRS-1, and ubiquitin (Figure 3). As reported previously, treatment with 120 *μ*M Cblin inhibited IRS-1 ubiquitination and interaction between Cbl-b and IRS-1 (Figure 3). Interestingly, Cblin-like synthetic peptide significantly inhibited Cbl-b-mediated ubiquitination of IRS-1 in a dose-dependent manner (Figure 3). Furthermore, Cblin-like synthetic peptide prevented the interaction between Cbl-b and IRS-1 (Figure 3). Densitometric analysis demonstrated that the IC_50_ value of this peptide was approximately 350 *μ*M (Figure 3), while the IC_50_ value of Cblin was approximately 120 *μ*M [4]. These results suggest that the Cblin-like peptide functioned as a Cbl-b inhibitor.

**Figure 4:**
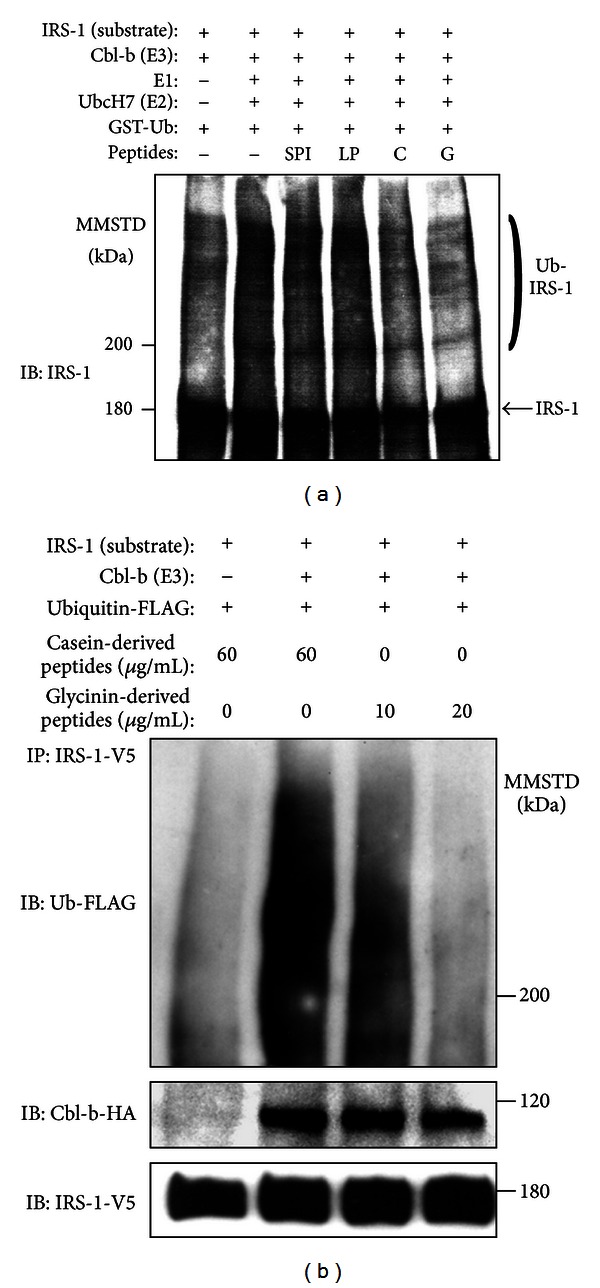
Inhibitory effect of soy-glycinin-derived peptides on Cbl-b-mediated IRS-1 ubiquitination. (a) Purified soy protein isolate (SPI), lipoprotein (LP), soy glycinin (G), and soy *β*-conglycinin (C) were digested with trypsin, then 20 *μ*g/mL of each of the hydrolysates was subjected to cell-free ubiquitination assay to elucidate their inhibitory effects on Cbl-b-mediated IRS-1 ubiquitination. (b) HEK293 cells transfected with mock vector/pCEFL-Cbl-b-HA, pcDNA3.1-rat IRS-1-V5, and pcDNA3-FLAG-Ubiquitin were treated with the indicated concentration of casein-derived (control) or soy-glycinin-derived peptides for 2 hours in the presence of 100 nM epoxomicin and 10 ng/mL IGF-1. Cell lysates from these cells were immunoprecipitated with an anti-V5 antibody. The immunoprecipitates were subjected to immunoblot (IB) analysis for the indicated proteins. MMSTD: molecular mass standards. Representative findings of three experiments with matching results.

Since soy glycinin contains Cblin-like peptide, we examined the inhibitory activities of various peptides (average length = 5-6 amino acids) derived from soy glycinin on Cbl-b-mediated ubiquitination of IRS-1. In our cell-free ubiquitination system, the interaction between Cbl-b and IRS-1 resulted in Cbl-b-dependent IRS-1 ubiquitination together with the expected electrophoretic pattern of ubiquitinated products of IRS-1 (Figure 4(a)). Among the various peptides employed, soy-glycinin-derived peptides significantly inhibited Cbl-b-mediated IRS-1 ubiquitination in the cell-free system, but soy-*β*-conglycinin-, LP-, and SPI-derived peptides did not (Figure 4(a)). We also examined the inhibitory effects of soy-glycinin-derived peptides using the cultured cell system. Consistent with the result of cell-free ubiquitination assay, soy-glycinin-derived peptides inhibited IRS-1 ubiquitination in Cbl-b-transfected HEK293 cells (Figure 4(b)). Furthermore, anti-IRS-1 immunoprecipitates from HEK293 cells treated with soy-glycinin-derived peptides contained smaller amounts of Cbl-b, compared to treatment with control peptide (Figure 4(b)). These results suggest that soy-glycinin-derived peptides significantly prevented Cbl-b-mediated IRS-1 ubiquitination through the suppression of Cbl-b and IRS-1 interaction.

### 3.3. Dietary Soy Glycinin Protein Prevents Denervation-Induced Muscle Wet Weight Loss

**Figure 5:**
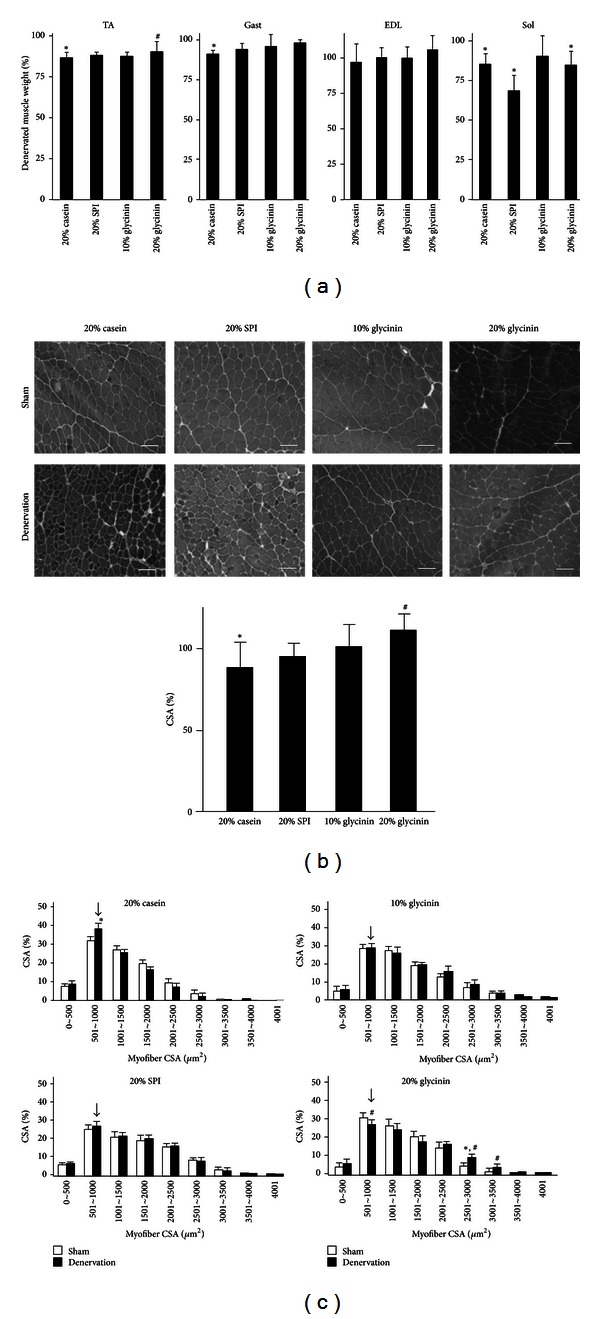
Effects of dietary soy glycinin protein on denervation-related decrease in muscle wet weight and muscle cross sectional area. The right and left legs of C57BL/6 mice were subjected to denervation and sham operation, respectively. Mice were divided at random into one of the following four diet groups: 20% casein group, 20% soy protein isolate group, 10% soy glycinin + 10% casein group (10% glycinin), and 20% soy glycinin group (20% glycinin). Each diet was started at 1 week before denervation and continued for the duration of the experiment period. Their hindlimb muscles were isolated at 4 days after denervation. (a) Wet weight of hindlimb muscles. Percent of denervated muscle weight was defined as the ratio of denervated hindlimb muscle wet weight to sham-operated hindlimb muscle wet weight in the mice. TA, tibialis anterior muscle; Gast, gastrocnemius muscle; EDL, extensor digitorum longus muscle; Sol, soleus muscle. Data are mean ± SD (*n* = 5). *P < 0.05, versus sham operated, ^#^ *P* < 0.05, versus 20% casein diet, by the Shirley-Williams' multiple comparison test. (b, c) Frozen sections of TA muscle were stained with H&E (b). Scale = 100 *μ*m. The cross sectional area (CSA) of myofibers was measured as described in Materials and Methods. The distribution of CSA in TA muscle was also shown (c). Percent of CSA indicates the ratio of the number of myofibers with the indicated area to the number of total myofibers in the section. The mean value from five individual sections was shown. Arrow indicates the most common value of CSA of denervated TA muscle. Solid bars: denervation group. Open bars: sham-operated group. Data are mean ± SD (*n* = 5). *P < 0.05, versus sham-operated, *P < 0.05, versus sham-operated, ^#^ *P* < 0.05, versus 20% casein diet, by the Shirley-Williams' multiple comparison test.

To examine the inhibitory effects of soy glycinin on Cbl-b-mediated muscle atrophy *in vivo*, the right leg muscles of C57BL/6J mice were denervated by sectioning the sciatic nerve while the left leg muscles of mice were sham operated. The denervated mice were divided into four groups based on the type of diet used: 20% casein diet group (*n* = 5), 20% SPI diet group (*n* = 5), 10% soy glycinin + 10% casein diet group (*n* = 5), and 20% soy glycinin diet group (*n* = 5). There were no significant differences in daily intake of food, protein, and energy among the four groups of mice (Table 3). In the denervated mice fed with 20% casein diet, the wet weight of denervated TA muscle decreased by 9% on 4 days after denervation, compared with sham-operated TA muscle (Figure 5(a)). In contrast, the SPI, 10% soy glycinin + 10% casein diet, and 20% soy glycinin diet prevented denervation-induced decrease in wet weight of TA muscle (Figure 5(a)). Interestingly, we also noted a dose-dependent inhibitory action of soy glycinin protein on denervation-induced wet TA muscle weight loss (Figure 5(a)). Similar inhibitory effects of soy glycinin on TA muscle were noted in gastrocnemius and EDL muscles (Figure 5(a)). However, changes in these muscles, besides TA muscle, were not statistically significant. Dietary soy proteins did not show an inhibitory effect on unloading-mediated muscle mass loss in soleus muscle (Figure 5(a)). Based on these results, we next examined the effects of dietary soy glycinin protein on denervation-mediated decrease in cross sectional area (CSA) of TA muscle. In mice of the 20% casein diet group, 4-day-denervation decreased the CSA of TA muscle by 10%, compared with sham operation (Figure 5(b)). SPI diet or 10% soy glycinin + 10% casein diet failed to restore the decrease in the CSA of denervated TA muscle (Figure 5(b)), whereas feeding with 20% soy glycinin diet significantly restored the CSA of denervated TA muscle to that of sham-operated TA muscle. Feeding with 20% soy glycinin diet also shifted the CSA distribution broader than that of mice of the SPI diet and 10% soy glycinin + 10% casein diet groups (Figure 5(c)).

### 3.4. Soy Glycinin Prevents Attenuation of IGF-1 Signaling in Denervated Muscle

**Figure 6:**
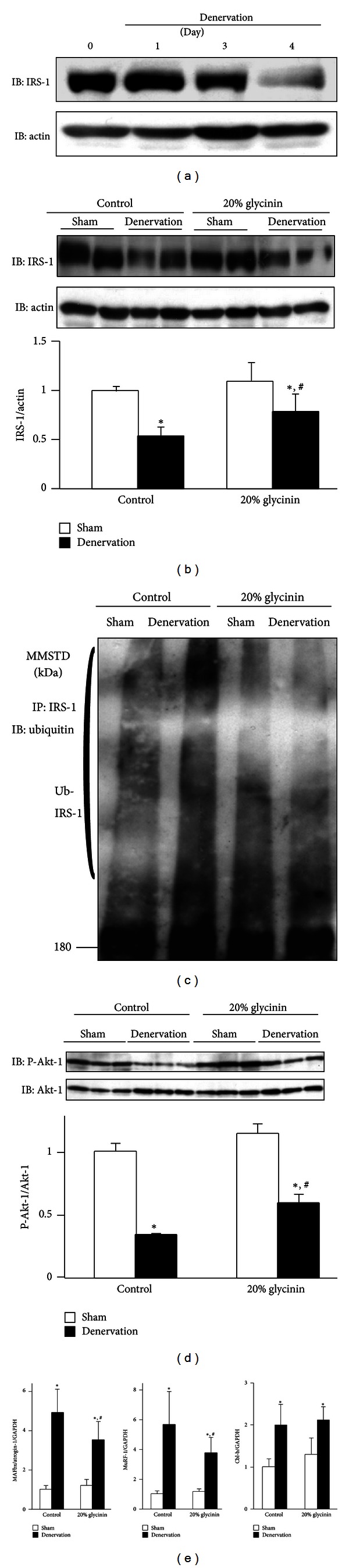
Effects of dietary soy glycinin protein on IGF-1 signaling in denervated muscle. The right and left legs of C57BL/6 mice were subjected to denervation and sham operation, respectively. The mice were fed with 20% casein (control) or 20% soy glycinin diet from 1 week before denervation till the end of the experiment. TA muscles were isolated at days 1, 3, 3.5, and 4 after denervation. (a) Homogenates of TA muscles isolated were subjected to immunoblotting (IB) for IRS-1 and actin on the indicated days after denervation. (b–d) Homogenates of TA muscles isolated from mice fed with control or 20% glycinin diet at 3.5 days after denervation were subjected to IB for IRS-1 and actin (b) and phosphorylated Akt-1 and total Akt-1 (d). The densitometric analysis of these images was performed with ImageJ software. Data are mean ± SD (*n* = 3). *P < 0.05 versus sham operation; ^#^ *P* < 0.05 versus control diet in denervated muscle. Immunoprecipitates (IP) from the TA muscles with an anti-IRS-1 antibody were subjected to IB for ubiquitin (c). Representative findings of five experiments with matching results. (e) Expression levels of MAFbx/atrogin-1, MuRF-1, Cbl-b, and GAPDH (internal control) transcripts in TA muscle analyzed by real-time RT-PCR. The level of each transcript was normalized to sham-operated mice fed with control diet. Data are mean ± SD (*n* = 5). *P < 0.05 versus sham operation; ^#^ *P* < 0.05 versus control diet in denervated muscle.

To address whether soy glycinin prevents Cbl-b-mediated degradation of IRS-1, IGF-1 signaling was estimated by immunoblotting in TA muscle of denervated mice of the control and 20% glycinin diet groups. The amount of IRS-1 in denervated TA muscle decreased in a time-dependent manner (Figure 6(a)). Furthermore, the degradation of IRS-1 in denervated TA muscle appeared at day 3 after denervation and was completed by day 4. Therefore, we examined the inhibitory effect of dietary soy glycinin protein on IRS-1 degradation using TA muscle after day 3.5 following denervation. The degradation and ubiquitination of IRS-1 were significantly prevented in mice of the 20% glycinin group, compared with the control group (Figures 6(b) and 6(c)). Based on these results, we also examined phosphorylation of Akt-1 in IGF-1 signaling. Consistent with IRS-1 protein level, the decrease in Akt-1 phosphorylation was smaller in the denervated TA muscle of the 20% glycinin group than in the control group (Figure 6(d)). Furthermore, denervation-induced expression of MAFbx/atrogin-1 and MuRF-1, but not Cbl-b, was abrogated in the denervated TA muscle of the 20% glycinin group, compared with the control group (Figure 6(e)). These results suggest that soy glycinin ameliorates denervation-induced decrease in IGF-1 signaling by suppressing IRS-1 degradation.

## 4. Discussion

We reported previously that Cbl-b interacts with and degrades IRS-1, an IGF-1 signaling intermediate, in skeletal muscles during unloading conditions [4], suggesting that Cbl-b is a key enzyme in unloading-related muscle atrophy. We also reported that the pentapeptide Cblin inhibited Cbl-b-mediated degradation of IRS-1, resulting in suppression of denervation-mediated muscle atrophy [4]. Therefore, we hypothesized that foodstuffs that contain Cblin-like amino acid sequence can be used to prevent unloading-induced muscle atrophy. The present study showed that the peptide “DIpYNP” contained in soy glycinin inhibited Cbl-b-mediated IRS-1 ubiquitination *in vitro*. Furthermore, dietary soy glycinin protein inhibited denervation-induced ubiquitination and degradation of IRS-1 *in vivo* and significantly prevented losses of wet weight and CSA of denervated TA muscle. Considered together, the present study suggests that soy glycinin is an effective protein source against unloading-related muscle atrophy possibly through diet-mediated inhibition of muscle ubiquitin ligase Cbl-b. Soy protein modulates protein turnover in skeletal muscles [5–7], although the mechanism of this beneficial effect is still unknown. The Cbl-bs inhibition of glycinin-derived peptide contributes, at least in part, to the beneficial effects of soy protein on muscle turnover.

Cbl-b conserves tyrosine kinase binding (TKB) domain, which is a modified Src homology 2 (SH2) domain and mediates binding in Cbl-b target molecules. Therefore, the dephosphorylated form of Cblin weakly interacted with the TKB domain of Cbl-b. In fact, the dephosphorylated Cblin peptide had less inhibitory activity on Cbl-b-mediated ubiquitination of IRS-1 than the phosphorylated one; the IC_50_ values of Cblin and dephosphorylated Cblin were approximately 120 *μ*M and 1 mM, respectively [4]. In the present study, phosphorylated Cblin-like peptide, DIpYNP, effectively inhibited Cbl-b-mediated ubiquitination and degradation of IRS-1, although we could not determine the IC_50_ value of dephosphorylated Cblin-like peptide. However, casein phosphopeptide (CPP) is a phosphorylated protein and resists dephosphorylation during digestion [12]. Furthermore, we noted that glycinin was remarkably phosphorylated, compared with egg white and bovine serum albumin proteins. Based on these findings, we suggest that Cblin-like sequence of dietary soy glycinin may be phosphorylated and absorbed without dephosphorylation. Further studies are necessary to elucidate the mechanism of the inhibitory effect of dietary soy glycinin *in vivo*.

We suggest that Cbl-b is a negative regulator of IGF-1 signaling during unloading [4]. Disturbed IGF-1 signaling, such as degradation of IRS-1, induces expression of MAFbx/atrogin-1 and MuRF-1 through dephosphorylation of Akt. Since unloading induces expression of Cbl-b at the mRNA level to ubiquitinate and degrade IRS-1 protein, it is consistent that expression of MAFbx/atrogin-1 and MuRF-1 was negatively regulated by dietary soy glycinin, but expression of Cbl-b was not, in our *in vivo *experiment.

Functional peptides are food-derived peptides that exert, beyond their nutritional value, physiological or hormone-like effect in humans. Functional peptides are inactive within the sequence of their parent protein and can be released by enzymatic hydrolysis either during gastrointestinal digestion or food processing. They are found in milk, egg, meat, and various kinds of fish as well as in many plants. Since amino acid sequence similar to Cblin in soy glycinin inhibited denervation-mediated ubiquitination of IRS-1, this sequence of soy is a potent functional peptide against muscle atrophy. Unlike various types of medicines, food-derived peptides have little side effects. Based on this property, dietary soy protein is a suitable protein source for efficient control of catabolism of muscle protein. In addition to soy protein, certain plants, for example, papaya and plum, contain proteins with amino acid sequence similar to Cblin. Unfortunately, they have only one Cblin-like sequence per molecule. Therefore, a high dose of functional foods is necessary to produce the inhibitory effect of ubiquitination *in vivo*. Global search for such food is necessary to develop more effective functional foods against muscle atrophy.

There is a controversy on the detection of food-derived bioactive peptides. The potential biological activities of peptides in food protein hydrolysates have frequently been screened by *in vitro* assays as shown in the present study. Unlike other functional substances, such as polyphenols and polyamines, some peptides in foods with *in vitro* biological activity might be further degraded by peptidases during the process of ingestion, digestion, and absorption. Consequently, these peptides might lose their potential activity detecting using in biological activity. Therefore, the amount of the peptides should be measured in their target organs through a feeding experiment. However, there are few papers showing the detection of food-derived peptides in target organs mainly due to the low concentration and interaction with plasma proteins. They suggest only that food-derived peptides could be accumulated in target organs, since they have a chronic rather than an acute effect on health. In the present study, we also failed to measure the quantity of Cblin-like peptide. Therefore, it is the next important subject. We are feeding mice a gene-modified protein, which contains high amount of the Cblin-like sequences, and are detecting the functional peptide in mouse portal vein. The detection of diet-derived Cblin-like peptides in muscle will be reported in our next paper.

## 5. Conclusion

The present study identified Cblin-like sequence, DI/FYNP, in soy glycinin. Similar to Cblin, treatment with soy glycinin prevented muscle atrophy in mice through the suppression of Cbl-b-induced IRS-1 degradation. The results also showed that 20% soy glycinin diet prevented denervation-related TA muscle wet weight loss in mice, suggesting that intake of soy glycinin could prevent muscle atrophy.

## Figures and Tables

**Table 1:** Composition of the different experimental diets used in the present study.

Product	Control diet	SPI* diet	10% soy glycinin diet	20% soy glycinin diet (g/100 g)
Casein	22.60	0	11.28	0
SPI*	0	22.40	0	0
Glycinin	0	0	11.47	22.89
*α*-Starch	44.44	44.55	44.36	44.27
Sucrose	22.23	22.29	22.19	22.15
Lard	4.23	4.24	4.22	4.21
Cellulose	2.00	2.01	1.99	1.99
Mineral mix	3.50	3.51	3.49	3.49
Vitamin mix	1.00	1.00	1.00	1.00

*SPI: soy protein isolate.

**Table 2:** Primers used for real-time RT-PCR.

Target gene		Sequence	Length (bp)
MAFbx/atrogin-1	S AS	5′-GGCGGACGGCTGGAA-3′ 5′-CAGATTCTCCTTACTGTATACCTCCTTGT-3′	100
MuRF-1	S AS	5′-ACGAGAAGAAGAGCGAGCTG-3′ 5′-CTTGGCACTTGAGAGAGGAAGG-3′	179
GAPDH	S AS	5′-CGTGTTCCTACCCCCAATGT-3′ 5′-ATGTCATCATACTTGGCAGGTTTCT-3′	74

AS: antisense primer; S: sense primer; MAFbx/atrogin-1: muscle atrophy F-box protein; MuRF-1: muscle-specific RING finger protein 1; GAPDH: glyceraldehyde-3-phosphate dehydrogenase.

**Table 3:** Daily intake of food, protein, and energy in mice.

	Control	SPI	10% glycinin	20% glycinin
Food intake (g/day)	4.78	4.31	4.20	4.17
Energy intake (kcal/day)	18.14	16.47	15.98	15.8
Protein intake (g/day)	0.91	0.82	0.80	0.79

## Abbreviations

ATP: Adenosine triphosphate
Atrogenes: Muscle atrophy-related genes
Cbl: Casitus b-lineage lymphoma
CSA: Cross sectional area
DMEM: Dulbecco's modified Eagle medium
EDL: Extensor digitorum longus muscle
FOXO3: Forkhead transcription factor-3
GAPDH: Glyceraldehyde-3-phosphate dehydrogenase
H&E: Hematoxylin-eosin staining
IB: Immunoblotting
IC_50_: 50% inhibitory concentration
IGF-1: Insulin-like growth factor-1
IRS-1: Insulin receptor substrate-1
LP: Lipoprotein
MAFbx/atrogin-1: Muscle atrophy F-box protein
MuRF-1: Muscle-specific RING finger protein 1
PCR: Polymerase chain reaction
SH2 domain: Src homology 2 domain
SPI: Soy protein isolate
TA muscle: Tibialis anterior muscle
TKB domain: Tyrosine kinase binding domain.
